# Rapamycin inhibits tamoxifen-induced endometrial proliferation in vitro as a pilot approach for endometrial protection in breast cancer

**DOI:** 10.1038/s41598-025-86586-8

**Published:** 2025-01-15

**Authors:** Akiko Nakamura, Yuji Tanaka, Tsukuru Amano, Akie Takebayashi, Akimasa Takahashi, Tetsuro Hanada, Yutaka Yoneoka, Shunichiro Tsuji, Takashi Murakami

**Affiliations:** Department of Obstetrics and Gynecology, Shiga University of Medical Science, 520-2192/Seta Tsukinowa-cho, Otsu, Shiga Japan

**Keywords:** Endometrial protection, Rapamycin, mTOR, Tamoxifen, Breast cancer, Cancer, Medical research, Molecular medicine, Oncology

## Abstract

Tamoxifen, a common adjuvant therapy for hormone receptor-positive breast cancer, is associated with an increased risk of endometrial pathologies, such as hyperplasia, polyps, and carcinoma. This study investigates rapamycin, an mTOR inhibitor, as a potential novel strategy for preventing tamoxifen-induced endometrial proliferation. This in vitro study utilised endometrial stromal cells isolated from infertile women. The cells were treated with tamoxifen alone or in combination with rapamycin, and proliferation was assessed using the CCK-8 assay. The activation of the mTOR pathway, as well as apoptosis and cell cycle markers, was evaluated by Western blotting to elucidate the molecular mechanisms underlying these effects. The study design emphasised simulating clinically relevant exposure levels. Tamoxifen significantly increased endometrial cell proliferation in a dose-dependent manner. Rapamycin effectively inhibited this proliferation, even at concentrations lower than those typically observed in clinical settings. Quantitative analysis by Western blotting showed activation of the mTOR pathway and cell cycle in the tamoxifen group, and inhibition of these pathways in the tamoxifen plus rapamycin combination group, whereas there was no change in apoptosis. In conclusion, rapamycin shows promise as a prophylactic agent against tamoxifen-induced endometrial pathologies, with potential implications for fertility preservation and endometrial protection in breast cancer patients.

**Supplementary Information:**

The online version contains supplementary material available at 10.1038/s41598-025-86586-8.

## Introduction

The primary regimen for adjuvant endocrine therapy in premenopausal patients with breast cancer includes the administration of tamoxifen for 5–10 years. In the United States, approximately 20,000 adolescent and young adult (AYA) women are diagnosed with breast cancer annually^1^, and approximately 70% of these cases are hormone receptor-positive^2^. Tamoxifen acts as an anti-oestrogen agent in breast tissue but has oestrogen-like effects on the endometrium and other gynaecological organs. Consequently, endometrial proliferation and hyperplasia occur in premenopausal patients treated with tamoxifen, increasing the risk of endometrial polyps, hyperplasia (with or without atypia), and carcinoma^3–5^. Endometrial polyps and hyperplasia can impair fertility owing to implantation failure^6,7^, making the control of tamoxifen-induced endometrial proliferation crucial for patients with AYA breast cancer desiring future fertility. Additionally, the prevention of endometrial carcinoma remains critical.

Currently, there is no high-level evidence supporting an effective method for endometrial protection in patients undergoing adjuvant tamoxifen therapy. The levonorgestrel intrauterine system (LNG-IUS) is the most extensively studied option; however, its efficacy is not consistently high and it has been associated with side effects, such as increased vaginal bleeding^8^. Moreover, its impact on breast cancer prognosis remains unclear^9,10^. Therefore, further research into drug-based strategies for endometrial protection in patients receiving adjuvant tamoxifen therapy is warranted.

Our investigation focused on the mTOR pathway and its inhibitor, rapamycin. mTOR inhibitors have been used clinically for over 20 years as post-transplantation immunosuppressants and antineoplastic agents for various malignancies, including breast cancer, and have demonstrated long-term safety. Studies have implicated tamoxifen in the pathogenesis of endometrial hyperplasia and carcinoma via activation of the mTOR pathway^11–13^, suggesting that targeting the mTOR pathway could mitigate tamoxifen-induced endometrial proliferation^14,15^. A randomised controlled trial also showed the efficacy of metformin in endometrial protection^16^, with one mechanism being the inhibition of the mTOR pathway^11^. However, experimental evidence using human-derived endometrial cells has not been reported. Therefore, in this study, we aimed to evaluate the efficacy of rapamycin in counteracting tamoxifen-induced endometrial proliferation in human endometrial cancer cells.

## Results

### Biopsies and characteristics of the Population

**Fig. 1:**
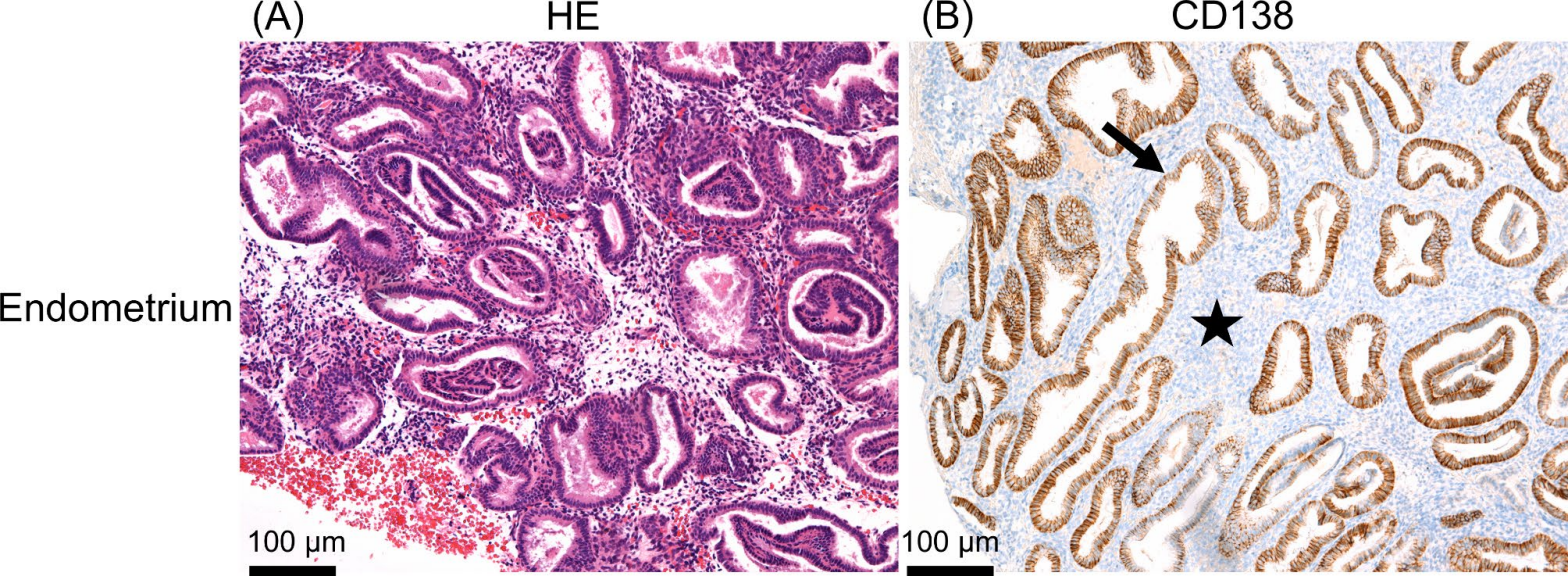
Characteristics of Endometrial Biopsies. (**A**) Haematoxylin-eosin staining of the endometrial biopsy specimen: Representative haematoxylin-eosin staining of the endometrial biopsy specimen. Histopathological evaluation revealed normal glandular and stromal architecture, with no evidence of excessive proliferation or atypia. The number and density of glands are appropriately maintained, and the gland-to-stroma ratio is within normal limits. These findings indicate the absence of endometrial hyperplasia. (**B**) CD138 immunostaining of the endometrial biopsy specimen: Representative CD-138 immunostaining images of endometrial biopsies. As the positive control, the glandular epithelium shows CD-138 positivity (indicated by an arrow), whereas the stroma (indicated by a star) shows no CD-138 positive cells. These findings indicate no signs of chronic endometritis.

The study population comprised six patients, from whom six biopsy specimens of the eutopic endometrium were obtained. All endometrial biopsy specimens were confirmed to be free of endometrial diseases such as endometritis and endometrial hyperplasia by haematoxylin-eosin (HE) staining and CD138 immunostaining (Fig. 1). The clinical characteristics of the study population are summarised in Table 1.

**Table 1:** Clinical characteristics of the included patients

	Infertile patients
Sample size	*n* = 6
Age (years)	37.5 (31–43) ^†^
Body mass index	23.3 (17.6–26.6) ^†^
Aetiology of infertility	Ovulatory dysfunction: *n* = 1 Male factor: *n* = 3 Unexplained infertility: *n* = 2
Number(s) of gravidity	0: *n* = 3 1: *n* = 2 2: *n* = 1
Number(s) of parity	0: *n* = 1 1: *n* = 5
Previous history of breast cancer	*n* = 0 (no history of taking tamoxifen)
Previous history of immune diseases	*n* = 0 (no history of taking immune suppressants)
Endometrial histopathology	Normal endometrium: *n* = 6 (Endometritis: *n* = 0) (Endometrial hyperplasia or neoplasia: *n* = 0)

^†^Data are presented as median (range).

### Tamoxifen exhibits a proliferative effect on endometrial stromal cells

**Fig. 2:**
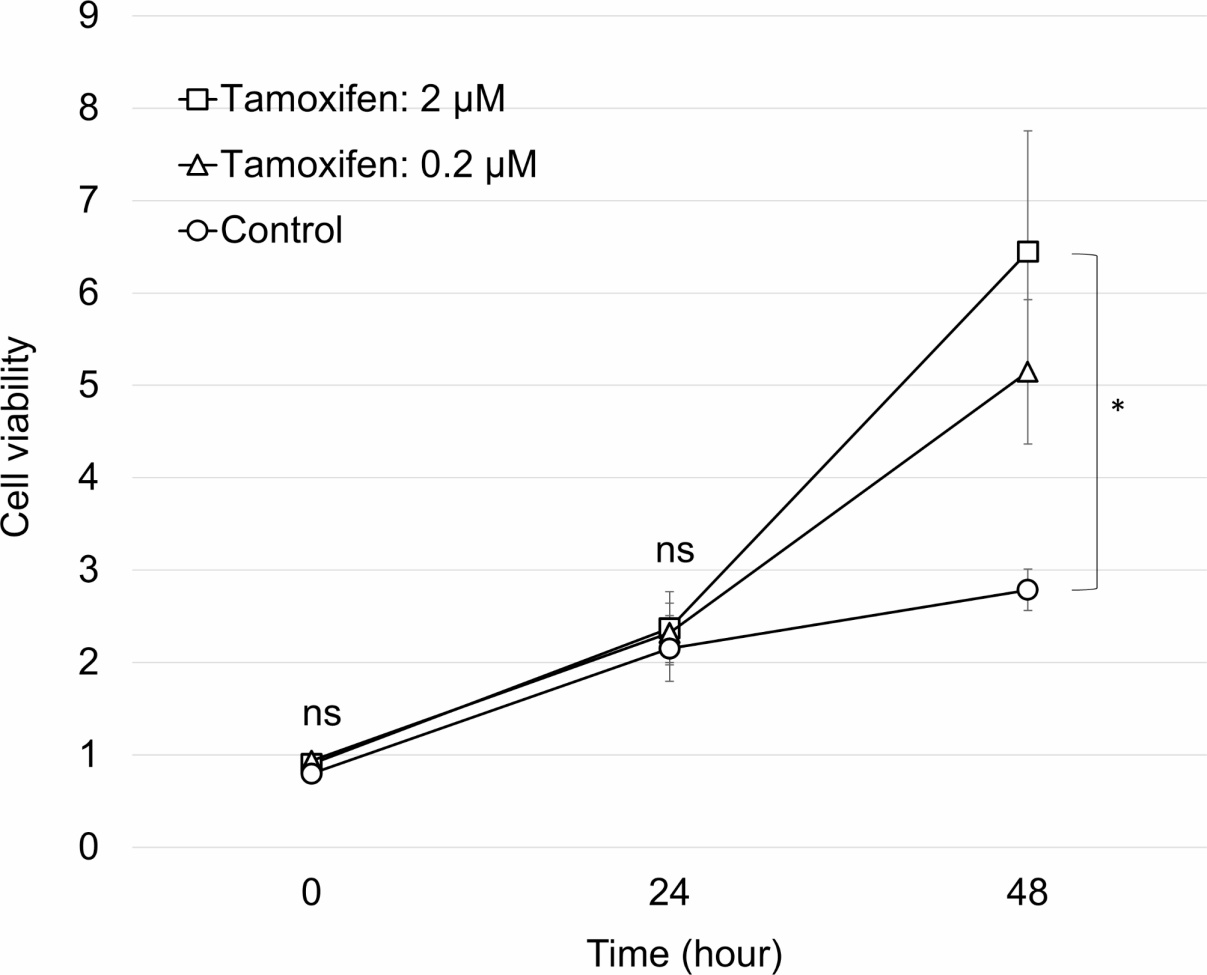
Proliferative effect of tamoxifen on endometrial stromal cells. The CCK-8 assay was used to assess the viability of endometrial stromal cells subjected to 0.2 µM or 2 µM tamoxifen treatment or no treatment. A significant increase in cell proliferation was observed only in the 2 µM of the tamoxifen group compared to the control group (*p < 0.05). All data are presented as the mean ± standard error. ns: not statistically significant.

The viabilities of endometrial stromal cells cultured without tamoxifen (control) or endometrial stromal cells cultured with low (0.2 µM) or high (2 µM) concentrations of tamoxifen were compared. Compared to the control group, after 48 h of culture, the viability of endometrial stromal cells was 1.8- and 2.3-fold higher in cultures with low (0.2 µM) and high (2 µM) concentrations of tamoxifen, respectively. A statistically significant difference was observed in the high-dose (2 µM) tamoxifen group compared to that in the control group but not in the low-dose (0.2 µM) group (*p* = 0.027 and *p* = 0.18, respectively). The low-dose (0.2 µM) concentration corresponded to clinically relevant serum concentrations, whereas the high-dose (2 µM) concentration reflected tissue-level concentrations. These findings demonstrate successful simulation of clinical conditions in an in vitro environment (Fig. 2).

### Rapamycin inhibits the proliferative effect of tamoxifen on endometrial stromal cells

**Fig. 3:**
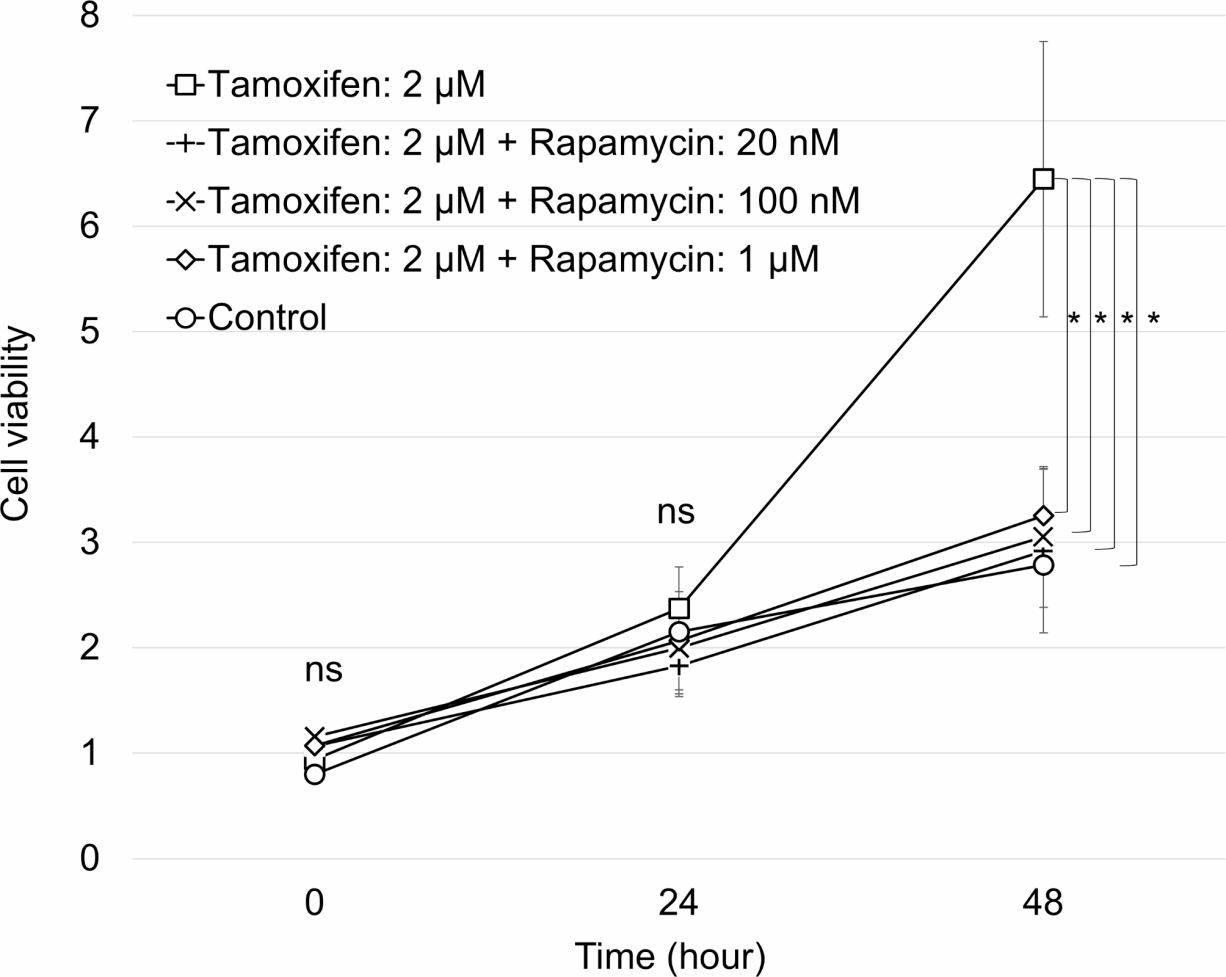
Impact of tamoxifen and rapamycin on endometrial stromal cell proliferation. The cell viability of endometrial stromal cells cultured under five conditions was compared (without the addition of tamoxifen or rapamycin [control], 2 µM of tamoxifen alone, and 2 µM of tamoxifen in combination with low [20 nM], medium [100 nM], and high [1 µM] concentrations of rapamycin). Rapamycin significantly inhibited tamoxifen-induced cell proliferation at all concentrations, with no significant differences among the rapamycin concentrations or between the control and tamoxifen-rapamycin groups. Statistical significance was observed only between the tamoxifen alone group and all other groups (*p < 0.05). All data are presented as the mean ± standard error. ns: Not statistically significant.

The viability of endometrial stromal cells cultured under five conditions was compared. Namely, without the addition of tamoxifen or rapamycin (control), high-dose (2 µM) tamoxifen alone, and high-dose tamoxifen in combination with low (20 nM), medium (100 nM), and high (1 µM) concentrations of rapamycin. A high dose of tamoxifen (2 µM) was used to mimic the tissue concentration of tamoxifen. A low concentration (20 nM) of rapamycin was designed to replicate clinical serum levels of rapamycin, whereas medium to high concentrations were used to replicate tissue concentrations of rapamycin. After 48 h of culture, the combination of high-dose (2 µM) tamoxifen with varying doses of rapamycin resulted in a 50% reduction in cell viability compared to that in the high-dose (2 µM) tamoxifen alone group. Rapamycin significantly inhibited the proliferative effects of tamoxifen on endometrial stromal cells at all concentrations tested. P values were 0.014 (Tamoxifen alone vs. control), 0.041 (Tamoxifen alone vs. Tamoxifen + High (1 µM) dose Rapamycin), 0.029 (Tamoxifen alone vs. Tamoxifen + Middle dose (100 nM) Rapamycin), and 0.022 (Tamoxifen alone vs. Tamoxifen + Low dose Rapamycin (20 nM)). No significant differences in cell proliferation were observed between the different rapamycin concentrations or between the control and any of the tamoxifen + rapamycin groups (Fig. 3).

### Morphology of endometrial stromal cells cultured with tamoxifen and Rapamycin

**Fig. 4:**
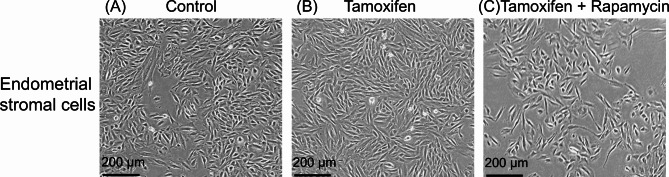
Endometrial stromal cells cultured with tamoxifen and rapamycin. Endometrial stromal cells cultured under three conditions: without tamoxifen and rapamycin (**A**: Control), with 2 µM tamoxifen (**B**: Tamoxifen), and with 2 µM of tamoxifen plus 100 nM rapamycin (**C**: Tamoxifen + Rapamycin) for 48 h. Scar bar: 200 μm.

Endometrial stromal cells were cultured under three conditions: without tamoxifen and rapamycin (control), with 2 µM tamoxifen (Tamoxifen), and with 2 µM tamoxifen + 100 nM rapamycin (Tamoxifen + Rapamycin). The cells were spindle-shaped, and no morphological differences were observed among the three groups. However, as shown by the CCK-8 assay, the tamoxifen group exhibited an increased cell number, whereas the tamoxifen + rapamycin group showed a reduced cell number (Fig. 4).

### Tamoxifen activates the mTOR pathway and cell cycle in cultured endometrial stromal cells, whereas rapamycin inhibits it

**Fig. 5:**
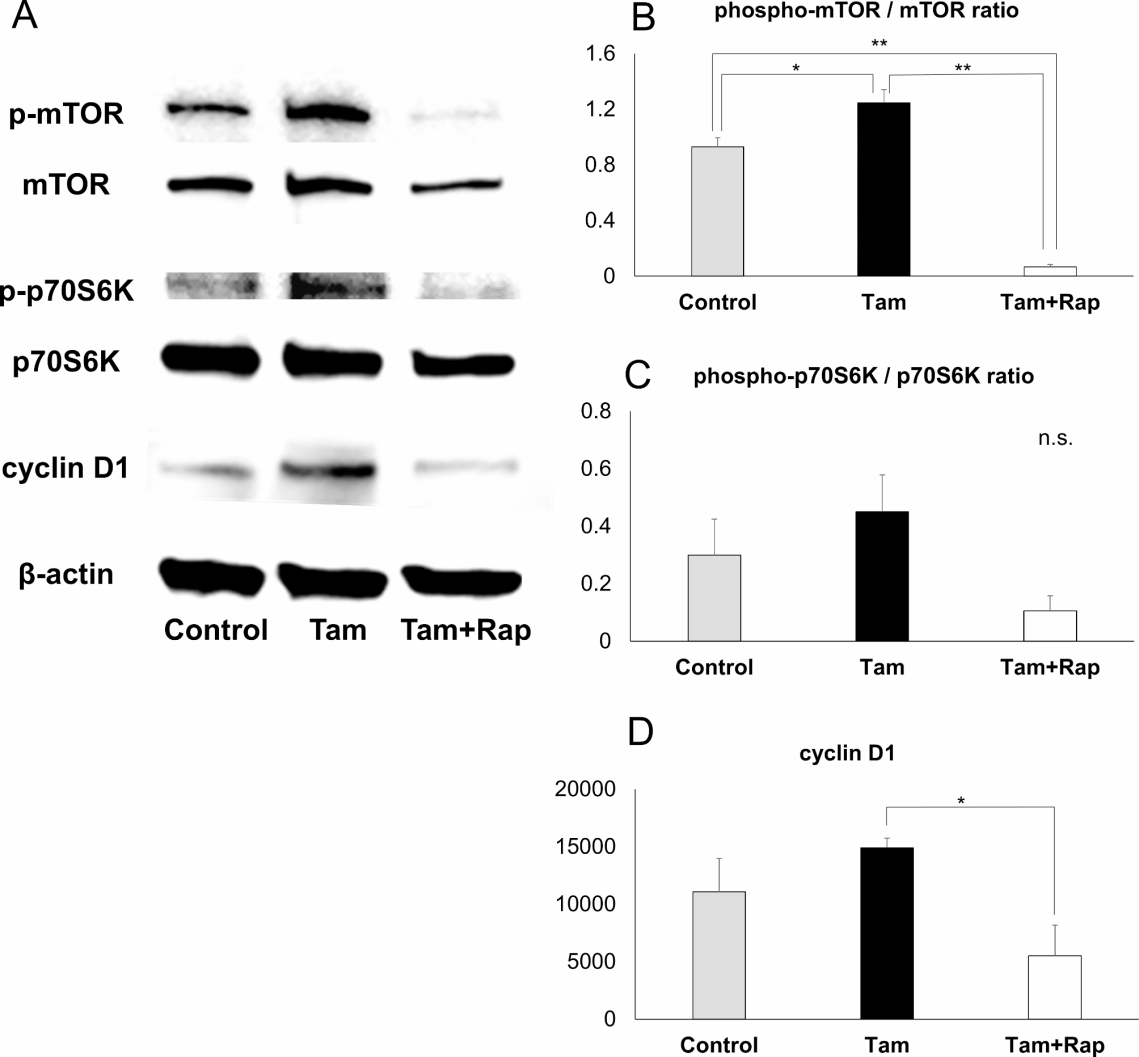
mTOR Pathway and Cell Proliferative Modulation in Cultured Endometrial Stromal Cells.

Endometrial stromal cells were cultured under three conditions: without tamoxifen and rapamycin (control), 2 µM tamoxifen (Tamoxifen), and 2 µM tamoxifen + 100 nM rapamycin (Tamoxifen + Rapamycin). Proteins from the resulting cell lysates were then used for Western blot analysis. The mTOR pathway and cell cycle in the cultured cells were assessed using Western blot analysis. The results showed that tamoxifen activated the phosphorylation of mTOR and its downstream effectors (p70S6K), whereas rapamycin inhibited the phosphorylation of mTOR and its downstream effectors. In addition, tamoxifen increased the expression of cyclin D1, whereas rapamycin suppressed its expression. There was a significant difference in the quantified phospho-mTOR/mTOR ratio in Western blot analysis between all groups (control vs. Tam, *p* = 0.022; control vs. Tam + Rap, *p* < 0.001; Tam vs. Tam + Rap, *p* < 0.001). In addition, there was a significant difference in the quantified cyclin D1 expression between the tamoxifen and tamoxifen + rapamycin groups (*p* = 0.045) (Fig. 5).

### Tamoxifen and rapamycin do not induce apoptosis in cultured endometrial stromal cells

**Fig. 6:**
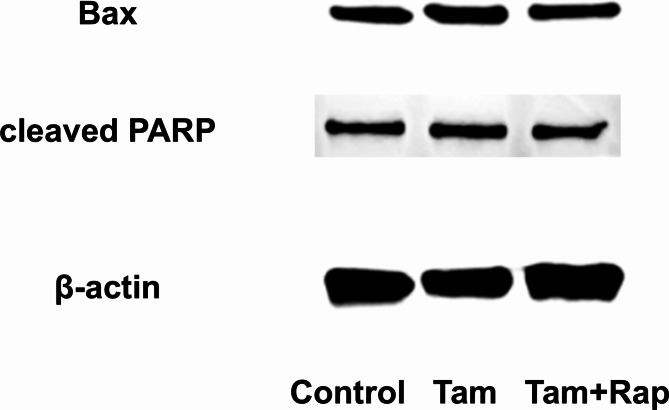
Evaluation of Apoptosis in Cultured Endometrial Stromal Cells.

Apoptosis in cultured endometrial stromal cells was assessed by Western blotting under conditions with or without tamoxifen and rapamycin. The results showed that tamoxifen and rapamycin did not alter the protein expression of Bax or cleaved PARP (Fig. 6).

## Discussion

In this study, we aimed to assess the protective effects of rapamycin against tamoxifen-induced endometrial pathologies, including hyperplasia, polyps, and cancer, in premenopausal patients with breast cancer. Our findings demonstrated for the first time that tamoxifen promotes the proliferation of endometrial cells through the activation of the mTOR pathway and that rapamycin inhibits tamoxifen-induced proliferation by inhibiting the mTOR pathway in an in vitro setting using human endometrial cells.

A significant strength of this study is the finding that rapamycin can inhibit the tamoxifen-induced proliferation of endometrial cells at concentrations that are substantially lower than those typically required in clinical practice. Notably, the inhibitory effect of rapamycin was not dose-dependent at higher concentrations, implying that lower doses may suppress tamoxifen-induced endometrial proliferation, thereby potentially minimising the associated adverse effects. It is critical to determine the appropriate concentration of rapamycin for endometrial protection, as mTOR inhibitors are known to induce dose-dependent side effects, including metabolic abnormalities and stomatitis when administered at clinical doses for antitumour purposes^17–20^. The efficacy of rapamycin at lower doses demonstrated in our study is particularly notable in this context. Previous studies, such as an in vivo study on everolimus^13^, also support the efficacy of mTOR inhibitors at reduced doses, with everolimus demonstrating effectiveness at 1.5 mg/kg, which is significantly below the 10 mg/kg dose typically used to replicate human clinical conditions^21^. Furthermore, the quasi-reversible binding of rapamycin to FK506-binding protein (FKBP)-12 in vivo may enhance its functional efficiency at lower doses than in vitro settings^22^.

This study further corroborates that tamoxifen-induced endometrial cell proliferation is mediated by mTOR pathway activation and that mTOR inhibitors effectively suppress this proliferation by inhibiting this pathway. The mTOR pathway plays a pivotal role in the regulation of cell cycle and proliferation and autophagy, cell adhesion, epithelial-mesenchymal transition, and drug resistance. Regarding cell cycle activation, we found that tamoxifen increased the expression of cyclin D1, whereas rapamycin decreased its expression in endometrial stromal cells. Rapamycin is suggested to inhibit the transition from the G1 to S phase by suppressing the PI3K/Akt/mTOR pathway and arresting the cell cycle at the G1 phase by reducing cyclin D1 synthesis. The involvement of the mTOR pathway in tamoxifen-induced endometrial hyperplasia and carcinoma has been reported in both clinical^12^ and basic research^13^ studies. Furthermore, numerous studies have shown that mTOR inhibitors are effective in treating endometrial hyperplasia and carcinoma^11,23–27^. In clinical trials, mTOR inhibitors have been combined with endocrine therapy to enhance their efficacy for the treatment of endometrial cancer, yielding favourable outcomes^28–30^. Despite these advances, studies specifically evaluating the efficacy of mTOR inhibitors in tamoxifen-induced endometrial disorders remain limited. The aforementioned in vivo study using everolimus demonstrated its potential to prevent tamoxifen-induced endometrial hyperplasia in mice^13^. However, its applicability in human-derived specimens remains unclear. This study aimed to bridge this gap by confirming these effects in human endometrial cells.

The mTOR inhibitors may provide additional therapeutic benefits beyond endometrial protection in patients with breast cancer receiving tamoxifen. Evidence suggests that mTOR inhibitors ameliorate tamoxifen resistance^13,31–34^, potentially enhancing the antitumour efficacy of tamoxifen. Furthermore, preclinical studies have indicated that mTOR inhibitors can mitigate age-related follicular loss^35–37^, which may contribute to preserving fertility in patients undergoing prolonged tamoxifen treatment. These additional benefits underscore the potential value of mTOR inhibitors such as rapamycin in comprehensive management strategies for tamoxifen therapy.

A limitation of this study was the use of endometrial stromal cells rather than epithelial cells, as the continuous culture of endometrial epithelial cells presents significant challenges. This substitution is common in endometrial research involving in vitro experiments^38^. Indeed, the literature on basic research related to endometrial hyperplasia includes studies examining treatments using human endometrial stromal cell lines^39,40^. However, recent advancements in organoid technology and co-culture systems have begun to facilitate the continuous culture of epithelial cells^41–45^. Therefore, future studies employing these advanced systems are highly desirable. Another limitation is the relatively small sample size. Nonetheless, we believe there is a scientific rationale for using clinical samples, even with a small sample size. This is because experiments using immortalised endometrial cell lines may not fully reflect the original characteristics of the cells because of the numerous modifications involved in creating these lines^46^. Considering these limitations, future research using clinical specimens with larger sample sizes, potentially in organoid or epithelial–stromal co-culture systems, would be highly beneficial.

**Fig. 7:**
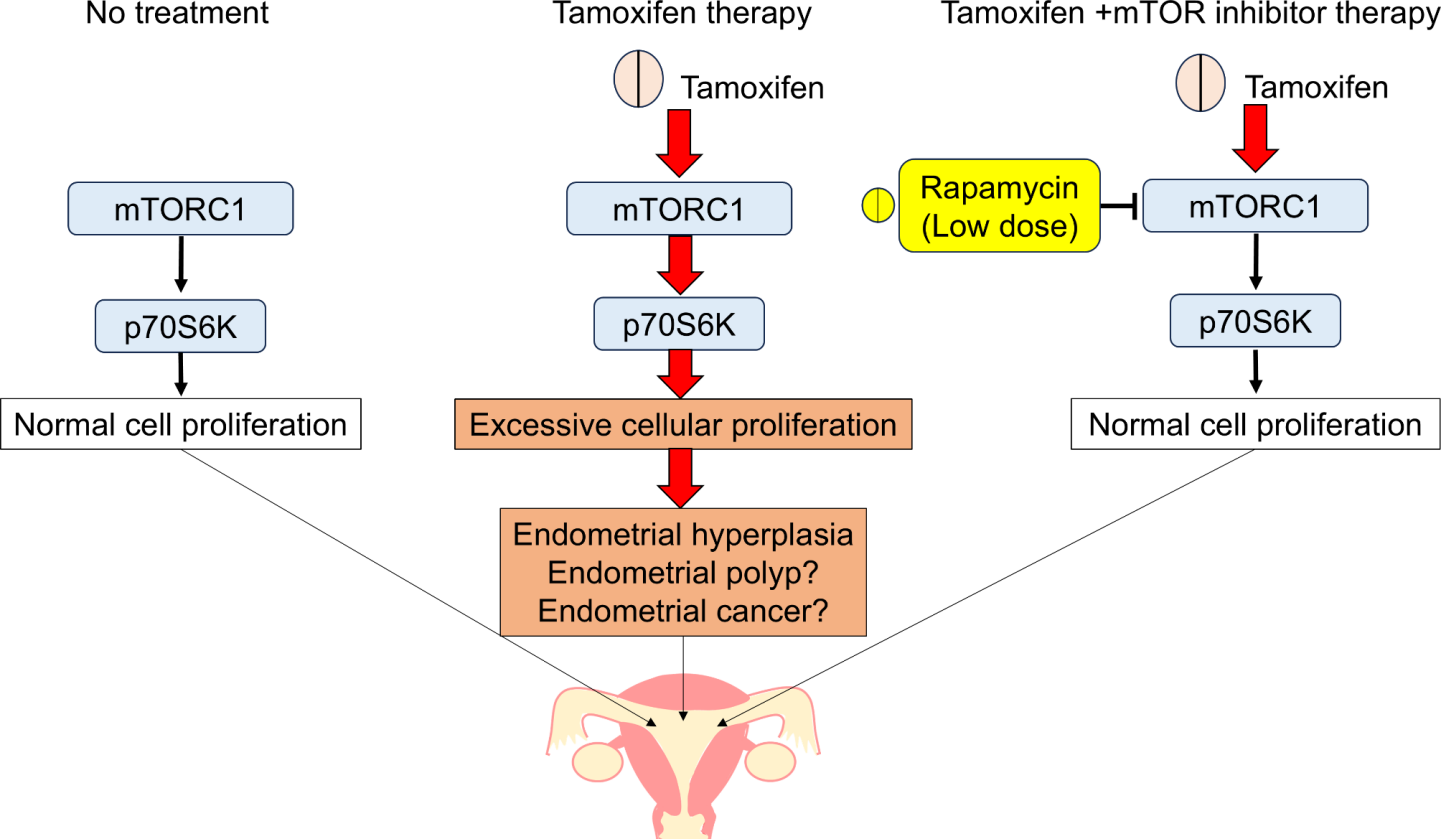
Summary of experimental findings. Tamoxifen enhanced endometrial cell proliferation via activation of the mTOR pathway, whereas mTOR inhibitors effectively suppressed tamoxifen-induced endometrial cell proliferation.

In conclusion, this in vitro study showed that rapamycin effectively inhibited tamoxifen-induced endometrial cell proliferation by targeting the mTOR pathway, even at concentrations lower than those typically used in clinical settings. These findings suggest that rapamycin is a promising prophylactic agent against tamoxifen-induced endometrial pathology (Fig. 7). Further research is needed to confirm the safety and efficacy of mTOR inhibitors, such as rapamycin, for endometrial protection in patients with breast cancer undergoing tamoxifen therapy.

## Methods

### Ethics declarations

All procedures involving human participants were conducted in accordance with the ethical standards of the institutional research committee and the 1964 Helsinki Declaration, as well as its later amendments. This study was approved by the Bioethics Committee of the Shiga University of Medical Science (Approval No. R2024-019). Written informed consent was obtained from all individual participants included in the study. Additionally, consent for publication was obtained from all participants.

### Sample collection

In this study, we used endometrial biopsy samples collected from patients undergoing infertility treatment via in vitro fertilisation and embryo transfer at our research institute to diagnose chronic endometritis, which is a known cause of implantation failure. Biopsies were performed during the menstrual cycle when the patients were not receiving hormone treatment. For each patient, half of the collected endometrial tissues were immersed in phosphate-buffered saline under sterile conditions and transported to the laboratory for cell culture. The other half of the endometrial tissue was fixed in formaldehyde, sectioned, and subjected to immunohistochemical staining using an anti-CD138 antibody (product number B-A38, Nichirei Corporation, Tokyo, Japan) and HE staining as previously described^47^. In this study, because endometritis has been reported to involve the mTOR pathway^14^, specimens that were negative for CD138 immunostaining (indicating no chronic endometritis) were used. In addition, only specimens from patients diagnosed as having no endometrial hyperplasia using, HE staining were used.

### Cell isolation and culture

Biopsy specimens were minced into small pieces, then digested with 0.2% collagenase (Sigma-Aldrich, St. Louis, MO) and 5‰ deoxyribonuclease for 1 h at 37 °C. After digestion, the same volume of Dulbecco’s Modified Eagle Medium/F12 (1:1) (Thermo Fisher Scientific, Waltham, MA) supplemented with 10% charcoal-treated foetal bovine serum (HyClone, GE Healthcare Life Sciences, Pittsburgh, PA, USA) was added to inactivate the collagenase. The epithelial cells were then removed using a 70-µm cell strainer, followed by centrifugation for 3 min at room temperature. After the pellet was resuspended, 2.2 × 10^6^ viable cells per dish were transferred to a 10-cm dish. The culture medium was changed every 2 or 3 days. Using immunocytochemistry for vimentin and cytokeratin, we confirmed that the purity of the endometrial stromal cells was > 95%. Endometrial stromal cells were used for subsequent experiments in a monolayer culture after one passage.

### Cell proliferation (CCK-8 assay)

Endometrial stromal cells were trypsinised, and 3 × 10^3^ cells were seeded per well in 96-well plates and cultured overnight. After 0, 24, and 48 h of treatment with tamoxifen and/or rapamycin, a Cell Counting Kit-8 (CCK-8; Dojindo Laboratories, Kumamoto, Japan) solution was added to each well and incubated at 37 °C for 1 h. A microplate reader was used to measure cell proliferation by measuring the absorbance value (OD) at 450 nm.

### Tamoxifen and rapamycin concentration settings and evidence for these settings

The serum concentration of tamoxifen in clinical settings is approximately 80–90 ng/mL, which is approximately 0.2 µM. Therefore, in this study, this concentration was defined as low-dose tamoxifen. Tissue concentrations in normal and breast cancer tissues range from 744 to 866 ng/mL, or approximately 2 µM^48,49^, which was used to define the high-dose tamoxifen setting.

For rapamycin, the effective serum concentration for immunosuppressive and antitumour purposes ranges from 5 to 20 ng/mL^50–52^, or approximately 20 nM, and this concentration was therefore set as the low-dose rapamycin concentration. Additionally, the commonly used concentration for in vitro antitumour effects in breast and endometrial cancer studies is 100 nM^53–55^, which we designated as the medium-dose rapamycin, a concentration below the expected tissue levels. Furthermore, the tissue concentrations of rapamycin in clinical and in vivo studies can be significantly higher than the serum concentrations, with differences of more than 40-fold^56,57^. Consequently, maximum tissue concentrations could theoretically reach approximately 1 µM in clinical settings, which was established as the high-dose rapamycin concentration in this study.

### Immunoblotting of cell lysates

Endometrial stromal cells were cultured with 2 µM of tamoxifen and/or 100 nM of rapamycin at 37 °C for 48 h before lysing in ice-cold RIPA buffer (Nacalai Tesque Inc., Kyoto, Japan). After scraping the lysate, it was centrifuged at 10,000 g at 4 °C for 10 min to obtain the supernatant, which was stored at − 80 °C until required for Western blotting.

Equal amounts of protein (5 µg) were loaded and separated by a 4–20% SDS-PAGE gel, before performing transfer and blocking. Polyvinylidene difluoride membranes (Bio-Rad Laboratories Inc., Richmond, CA) were incubated with the following primary antibodies at 4 °C overnight: phospho-mTOR (#2971; 1:1000), mTOR (#2983; 1:1000), phospho-p70S6K (#9205; 1:1000), p70S6K (#2708; 1:1000), cyclin D1 (#2978; 1:1000), Bax (#5023; 1:1000), cleaved PARP (#9541; 1:1000), and β-actin (#4970; 1:10000), all from Cell Signalling Technology (Danvers, MA) diluted in Can Get Signal (Toyobo Co Ltd., Osaka, Japan). Horseradish peroxidase-conjugated goat anti-rabbit IgG (#7074, CST) was used to detect the proteins, and a Chemi-Lumi One Super kit (Nacalai Tesque Inc.) was used to visualise the bands. β-actin was used as a housekeeping protein. Image J (National Institutes of Health, USA) was used to quantify the ratios of phospho-mTOR/mTOR and phospho-p70S6K/p70S6K.

### Statistical analysis

The results were analysed using JMP version 17 software (JMP, USA). A one-way analysis of variance (ANOVA) was used where appropriate. Data are presented as the mean ± SEM. Differences among groups were tested using ANOVA. If an overall significant difference was found, a post hoc Tukey’s test was performed. Statistical significance was set at *p* < 0.05.

## Electronic supplementary material

Below is the link to the electronic supplementary material.

Supplementary Material 1

Supplementary Material 2

## Data Availability

The data are available in the article and, when necessary, by request from yujit@belle.shiga-med.ac.jp.

## References

[1] Trivers, K. F. et al. Estimates of young breast cancer survivors at risk for infertility in the U.S. *Oncologist* **19**, 814–822 (2014). EPub. PMID: 24951610, PMCID: PMC4122477. 24951610 10.1634/theoncologist.2014-0016 PMC4122477

[2] Keegan, T. H. M., DeRouen, M. C., Press, D. J., Kurian, A. W. & Clarke, C. A. Occurrence of breast cancer subtypes in adolescent and young adult women. *Breast Cancer Res.* **14**, R55 (2012). PMID: 22452927, PMCID: PMC3446389. 22452927 10.1186/bcr3156 PMC3446389

[3] Lee, M., Piao, J. & Jeon, M. J. Risk factors associated with endometrial pathology in premenopausal breast cancer patients treated with tamoxifen. *Yonsei Med. J.* **61**, 317–322 (2020). PMID: 32233174, PMCID: PMC7105402. 32233174 10.3349/ymj.2020.61.4.317 PMC7105402

[4] Chalas, E. et al. Benign gynecologic conditions among participants in the Breast Cancer Prevention Trial. *Am. J. Obstet. Gynecol.* 192, 1230–1237; discussion 1237–1239 PMID: 15846210. (2005). 10.1016/j.ajog.2004.12.083 15846210

[5] Ryu, K. J. et al. Risk of endometrial polyps, hyperplasia, carcinoma, and uterine cancer after tamoxifen treatment in premenopausal women with breast cancer. *JAMA Netw. Open.* **5**, e2243951 (2022). PMID: 36441547, PMCID: PMC9706361. 36441547 10.1001/jamanetworkopen.2022.43951 PMC9706361

[6] An, H. et al. Pregnancy outcomes in infertile patients with endometrial hyperplasia with or without atypia undergoing in vitro fertilization: the early-follicular long protocol is superior to midluteal long protocol. *Front. Endocrinol. (Lausanne)* **15**, 1314432 (2024), PMID: 38449849, PMCID: PMC10916507. 10.3389/fendo.2024.1314432 PMC10916507 38449849

[7] Pérez-Medina, T. et al. Endometrial polyps and their implication in the pregnancy rates of patients undergoing intrauterine insemination: a prospective, randomized study. *Hum. Reprod.* **20**, 1632–1635 (2005). EPub. PMID: 15760959. 15760959 10.1093/humrep/deh822

[8] Romero, S. A., Young, K., Hickey, M. & Su, H. I. Levonorgestrel intrauterine system for endometrial protection in women with breast cancer on adjuvant tamoxifen. *Cochrane Database Syst. Rev.* **12**, CD007245 (2020). PMID: 33348436, PMCID: PMC8092675. 33348436 10.1002/14651858.CD007245.pub4 PMC8092675

[9] Zürcher, A., Knabben, L., Janka, H. & Stute, P. Influence of the levonorgestrel-releasing intrauterine system on the risk of breast cancer: a systematic review. *Arch. Gynecol. Obstet.* **307**, 1747–1761 (2023). EPub 2022 Jun 18. PMID: 35716207, PMCID: PMC10147797. 35716207 10.1007/s00404-022-06640-y PMC10147797

[10] Conz, L. et al. Levonorgestrel-releasing intrauterine system and breast cancer risk: a systematic review and meta-analysis. *Acta Obstet. Gynecol. Scand.* **99**, 970–982 (2020). EPub. PMID: 31990981. 31990981 10.1111/aogs.13817

[11] Erdemoglu, E., Güney, M., Giray, S. G., Take, G. & Mungan, T. Effects of metformin on mammalian target of rapamycin in a mouse model of endometrial hyperplasia. *Eur. J. Obstet. Gynecol. Reprod. Biol.* 145, 195–199 EPub. PMID: 19501448. (2009). 10.1016/j.ejogrb.2009.04.034 19501448

[12] Tergas, A. I. et al. Clinico-pathologic comparison of type II endometrial cancers based on tamoxifen exposure. *Gynecol. Oncol.* **127**, 316–320 (2012). EPub. PMID: 22835717. 22835717 10.1016/j.ygyno.2012.07.105

[13] Erdemoglu, E., Güney, M., Take, G., Giray, S. G. & Mungan, T. RAD001 (everolimus) Can prevent tamoxifen-related endometrial and stromal hyperplasia. *Int. J. Gynecol. Cancer* 19, 375–379 PMID: 19407562. (2009). 10.1111/IGC.0b013e3181a1a334 19407562

[14] Driva, T. S., Schatz, C., Sobočan, M. & Haybaeck, J. The role of mTOR and eIF signaling in benign endometrial diseases. *Int. J. Mol. Sci.* **23**, 3416 (2022), PMID: 35408777, PMCID: PMC8998789. 10.3390/ijms23073416 PMC8998789 35408777

[15] Wang, B., Gao, M., Yao, Y., Li, H. & Zhang, X. Focusing on the role of protein kinase mTOR in endometrial physiology and pathology: insights for therapeutic interventions. *Mol. Biol. Rep.* 51, 359 PMID: 38400863. (2024). 10.1007/s11033-023-08937-w 38400863

[16] Davis, S. R. et al. The benefits of adding metformin to tamoxifen to protect the endometrium-A randomized placebo-controlled trial. *Clin. Endocrinol. (Oxf)*. **89**, 605–612 (2018). EPub. PMID: 30107043. 30107043 10.1111/cen.13830

[17] Iacovelli, R. et al. Incidence and risk of pulmonary toxicity in patients treated with mTOR inhibitors for malignancy. A meta-analysis of published trials. *Acta Oncol.* **51**, 873–879 (2012). EPub. PMID: 22909392. 22909392 10.3109/0284186X.2012.705019

[18] Yuan, R., Kay, A., Berg, W. J. & Lebwohl, D. Targeting tumorigenesis: development and use of mTOR inhibitors in cancer therapy. *J. Hematol. Oncol.* **2**, 45 (2009), PMID: 19860903, PMCID: PMC2775749. 10.1186/1756-8722-2-45 PMC2775749 19860903

[19] Kwon, Y. Mechanism-based management for mucositis: option for treating side effects without compromising the efficacy of cancer therapy. *Onco Targets Ther.* **9**, 2007–2016 (2016). PMID: 27103826, PMCID: PMC4827894. 27103826 10.2147/OTT.S96899 PMC4827894

[20] Kaplan, B., Qazi, Y. & Wellen, J. R. Strategies for the management of adverse events associated with mTOR inhibitors. *Transpl. Rev. (Orlando)*. **28**, 126–133 (2014). EPub. PMID: 24685370. 10.1016/j.trre.2014.03.002 24685370

[21] Bradshaw-Pierce, E. L. et al. Utilization of quantitative in vivo pharmacology approaches to assess combination effects of everolimus and irinotecan in mouse xenograft models of colorectal cancer. *PLOS One*. **8**, e58089 (2013). EPub. PMID: 23520486, PMCID: PMC3592886. 23520486 10.1371/journal.pone.0058089 PMC3592886

[22] Wymann, M. P. & Borsari, C. Two-drug trick to target the brain blocks toxicity in the body. *Nature* 609, 681–683 PMID: 36104488. (2022). 10.1038/d41586-022-02892-5 36104488

[23] Milam, M. R. et al. Reduced progression of endometrial hyperplasia with oral mTOR inhibition in the Pten heterozygote murine model. *Am. J. Obstet. Gynecol.* 196, 247.e1-247.e5 PMID: 17346540. (2007). 10.1016/j.ajog.2006.10.872 17346540

[24] Bajwa, P. et al. Overactive mTOR signaling leads to endometrial hyperplasia in aged women and mice. *Oncotarget* **8**, 7265–7275 (2017). PMID: 27980219, PMCID: PMC5352319. 27980219 10.18632/oncotarget.13919 PMC5352319

[25] Wang, Y., Zhu, L., Kuokkanen, S. & Pollard J.W. Activation of protein synthesis in mouse uterine epithelial cells by estradiol-17β is mediated by a PKC-ERK1/2-mTOR signaling pathway. *Proc. Natl. Acad. Sci. U S A*. **112**, E1382–E1391 (2015). EPub. PMID: 25733860, PMCID: PMC4371960. 25733860 10.1073/pnas.1418973112 PMC4371960

[26] Sahoo, S. S. et al. Adipose-derived VEGF-mTOR signaling promotes endometrial hyperplasia and cancer: implications for obese women. *Mol. Cancer Res.* **16**, 309–321 (2018). EPub 2017 Nov 13. PMID: 29133593. 29133593 10.1158/1541-7786.MCR-17-0466

[27] Li, X. et al. Reversing the reduced level of endometrial GLUT4 expression in polycystic ovary syndrome: a mechanistic study of metformin action. *Am. J. Transl Res.* **7**, 574–586 (2015). PMID: 26045896, PMCID: PMC4448196. 26045896 PMC4448196

[28] Slomovitz, B. M. et al. Phase II study of everolimus and letrozole in patients with recurrent endometrial carcinoma. *J. Clin. Oncol.* **33**, 930–936 (2015). EPub. PMID: 25624430, PMCID: PMC4348638. 25624430 10.1200/JCO.2014.58.3401 PMC4348638

[29] Heudel, P. et al. Safety and efficacy of the mTOR inhibitor, Vistusertib, combined with anastrozole in patients with hormone receptor-positive recurrent or metastatic endometrial cancer: the Victoria Multicenter, open-label, phase 1/2 randomized clinical trial. *JAMA Oncol.* **8**, 1001–1009 (2022). PMID: 35551299, PMCID: PMC9100474. 35551299 10.1001/jamaoncol.2022.1047 PMC9100474

[30] Soliman, P. T. et al. Everolimus, letrozole, and metformin in women with advanced or recurrent endometrioid endometrial cancer: a multi-center, single arm, phase II study. *Clin. Cancer Res.* **26**, 581–587 (2020). EPub 2019 Oct 18. PMID: 31628143, PMCID: PMC7002216. 31628143 10.1158/1078-0432.CCR-19-0471 PMC7002216

[31] deGraffenried, L. A. et al. Inhibition of mTOR activity restores tamoxifen response in breast cancer cells with aberrant akt activity. *Clin. Cancer Res.* **10**, 8059–8067 (2004). PMID: 15585641. 15585641 10.1158/1078-0432.CCR-04-0035

[32] Bostner, J. et al. Activation of akt, mTOR, and the estrogen receptor as a signature to predict tamoxifen treatment benefit. *Breast Cancer Res. Treat.* **137**, 397–406 (2013). EPub 2012 Dec 15. PMID: 23242584, PMCID: PMC3539073. 23242584 10.1007/s10549-012-2376-y PMC3539073

[33] Bachelot, T. et al. Randomized phase II trial of everolimus in combination with tamoxifen in patients with hormone receptor-positive, human epidermal growth factor receptor 2-negative metastatic breast cancer with prior exposure to aromatase inhibitors: a GINECO study. *J. Clin. Oncol.* **30**, 2718–2724 (2012). EPub. PMID: 22565002. 22565002 10.1200/JCO.2011.39.0708

[34] Sánchez-Bayona, R. et al. Everolimus plus endocrine therapy beyond CDK4/6 inhibitors progression for HR+ /HER2- advanced breast cancer: a real-world evidence cohort. *Breast Cancer Res. Treat.* **206**, 551–559 (2024). EPub. PMID: 38703285. 38703285 10.1007/s10549-024-07324-8

[35] Zhang, X. M. et al. Rapamycin preserves the follicle pool reserve and prolongs the ovarian lifespan of female rats via modulating mTOR activation and sirtuin expression. *Gene* 523, 82–87 EPub. PMID: 23566837. (2013). 10.1016/j.gene.2013.03.039 23566837

[36] Yang, H. et al. Characterization of female germline stem cells from adult mouse ovaries and the role of rapamycin on them. *Cytotechnology* **70**, 843–854 (2018). EPub. PMID: 29372468, PMCID: PMC5851976. 29372468 10.1007/s10616-018-0196-6 PMC5851976

[37] Sato, Y. & Kawamura, K. Rapamycin treatment maintains developmental potential of oocytes in mice and follicle reserve in human cortical fragments grafted into immune-deficient mice. *Mol. Cell. Endocrinol.* **504**, 110694 (2020). EPub 2019 Dec 27. PMID: 31887337. 31887337 10.1016/j.mce.2019.110694

[38] Riaz, M. et al. (ed, A.) Long-term maintenance of viable human endometrial epithelial cells to analyze estrogen and progestin effects. *Cells* **13** 811 (2024). PMID: 38786035, PMCID: PMC11120542. 38786035 10.3390/cells13100811 PMC11120542

[39] Stanojevic-Pirkovic, M. et al. UV irradiation induces apoptosis in the human endometrial stromal cell line (ThESC). *J. BUON*. **25**, 1541–1546 (2020). 32862602

[40] Nikolic, I. et al. Induction of mitochondrial apoptotic pathway by raloxifene and estrogen in human endometrial stromal ThESC cell line. *Arch. Med. Sci.* **13**, 293–301 (2017). 28261281 10.5114/aoms.2016.59874 PMC5332444

[41] James, D. G., Graham, E. & Hamblin, A. Immunology of multisystem ocular disease. *Surv. Ophthalmol.* 30, 155–167 PMID: 3878603. (1985). 10.1016/0039-6257(85)90059-1 3878603

[42] Yokomizo, R. et al. Endometrial regeneration with endometrial epithelium: homologous orchestration with endometrial stroma as a feeder. *Stem Cell. Res. Ther.* **12**, 130 (2021). PMID: 33579355, PMCID: PMC7881492. 33579355 10.1186/s13287-021-02188-x PMC7881492

[43] Boretto, M. et al. Development of organoids from mouse and human endometrium showing endometrial epithelium physiology and long-term expandability. *Development* 144, 1775–1786 EPub. PMID: 28442471. (2017). 10.1242/dev.148478 28442471

[44] Turco, M. Y. et al. Long-term, hormone-responsive organoid cultures of human endometrium in a chemically defined medium. *Nat. Cell. Biol.* **19**, 568–577 (2017). EPub. PMID: 28394884, PMCID: PMC5410172. 28394884 10.1038/ncb3516 PMC5410172

[45] Fitzgerald, H. C., Dhakal, P., Behura, S. K., Schust, D. J. & Spencer, T. E. Self-renewing endometrial epithelial organoids of the human uterus. *Proc. Natl. Acad. Sci. U S A*. **116**, 23132–23142 (2019). EPub. PMID: 31666317, PMCID: PMC6859318. 31666317 10.1073/pnas.1915389116 PMC6859318

[46] Chalak, M. et al. Cell immortality: in vitro effective techniques to achieve and investigate its applications and challenges. *Life (Basel)*. **14**, 417 (2024). 38541741 10.3390/life14030417 PMC10971253

[47] Wu, D. et al. Chronic endometritis modifies decidualization in human endometrial stromal cells. *Reprod. Biol. Endocrinol.* **15**, 16 (2017). PMID: 28259137, PMCID: PMC5336610. 28259137 10.1186/s12958-017-0233-x PMC5336610

[48] Kisanga, E. R. et al. Tamoxifen and metabolite concentrations in serum and breast cancer tissue during three dose regimens in a randomized preoperative trial. *Clin. Cancer Res.* 10, 2336–2343 PMID: 15073109. (2004). 10.1158/1078-0432.ccr-03-0538 15073109

[49] Lien, E. A. et al. Serum concentrations of tamoxifen and its metabolites increase with age during steady-state treatment. *Breast Cancer Res. Treat.* **141**, 243–248 (2013). EPub. PMID: 23996142, PMCID: PMC3785179. 23996142 10.1007/s10549-013-2677-9 PMC3785179

[50] Stenton, S. B., Partovi, N. & Ensom, M. H. H. Sirolimus: the evidence for clinical pharmacokinetic monitoring. *Clin. Pharmacokinet.* **44**, 769–786 (2005). PMID: 16029064. 16029064 10.2165/00003088-200544080-00001

[51] MacDonald, A., Scarola, J., Burke, J. T. & Zimmerman, J. J. Clinical pharmacokinetics and therapeutic drug monitoring of sirolimus. *Clin. Ther.* 22 Suppl B, B101-B121 PMID: 10823378. (2000). 10.1016/s0149-2918(00)89027-x 10823378

[52] Mahalati, K. & D Kahan, B. Clinical pharmacokinetics of sirolimus. *Clin. Pharmacokinet.* **40**, 573–585 (2001). PMID: 11523724. 11523724 10.2165/00003088-200140080-00002

[53] Liu, T. et al. Combinatorial effects of lapatinib and rapamycin in triple-negative breast cancer cells. *Mol. Cancer Ther.* **10**, 1460–1469 (2011). EPub. PMID: 21690228, PMCID: PMC4908959. 21690228 10.1158/1535-7163.MCT-10-0925 PMC4908959

[54] Noh, W. C. et al. Determinants of rapamycin sensitivity in breast cancer cells. *Clin. Cancer Res.* 10, 1013–1023 PMID: 14871980. (2004). 10.1158/1078-0432.ccr-03-0043 14871980

[55] Zhou, W. J. et al. Rapamycin synergizes with cisplatin in antiendometrial cancer activation by improving IL-27-stimulated cytotoxicity of NK cells. *Neoplasia* **20**, 69–79 (2018). EPub 2017 Dec 1. PMID: 29195127, PMCID: PMC5724748. 29195127 10.1016/j.neo.2017.11.003 PMC5724748

[56] Napoli, K. L., Wang, M. E., Stepkowski, S. M. & Kahan, B. D. Distribution of sirolimus in rat tissue. *Clin. Biochem.* 30, 135–142 PMID: 9127695. (1997). 10.1016/s0009-9120(96)00157-9 9127695

[57] Kuhn, J. G. et al. Pharmacokinetic and tumor distribution characteristics of temsirolimus in patients with recurrent malignant glioma. *Clin. Cancer Res.* **13**, 7401–7406 (2007). PMID: 18094423, PMCID: PMC4918812. 18094423 10.1158/1078-0432.CCR-07-0781 PMC4918812

