# Supplementary figures and images for: Rapamycin inhibits tamoxifen-induced endometrial proliferation in vitro as a pilot approach for endometrial protection in breast cancer
Source: Sci Rep. 2025 Jan 15;15:2112. doi: 10.1038/s41598-025-86586-8 (PMC11739499; doi:10.1038/s41598-025-86586-8)

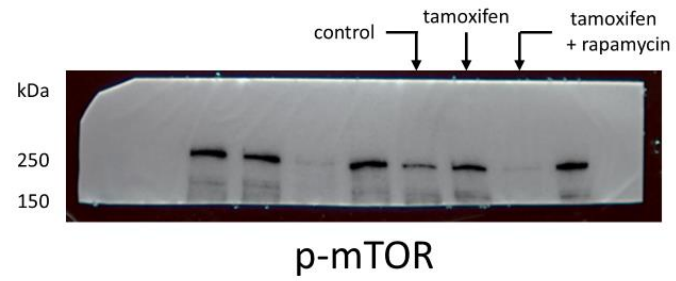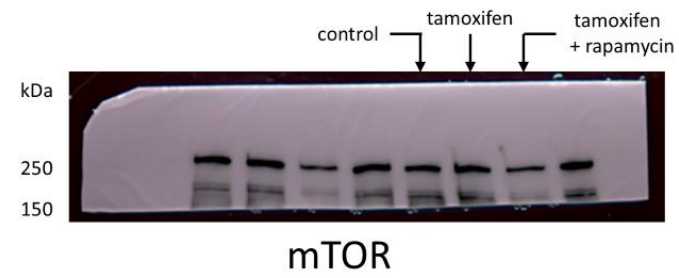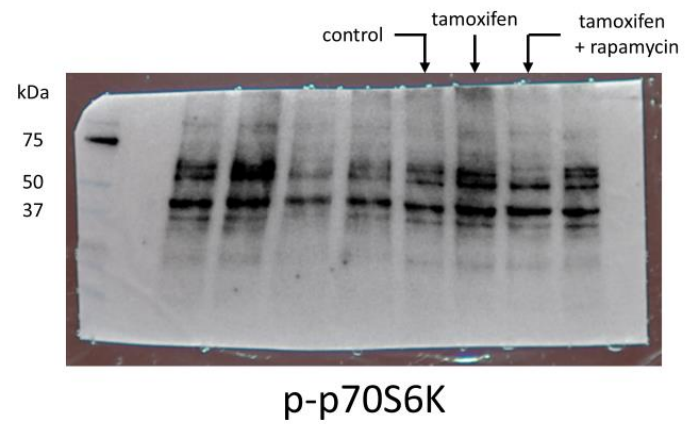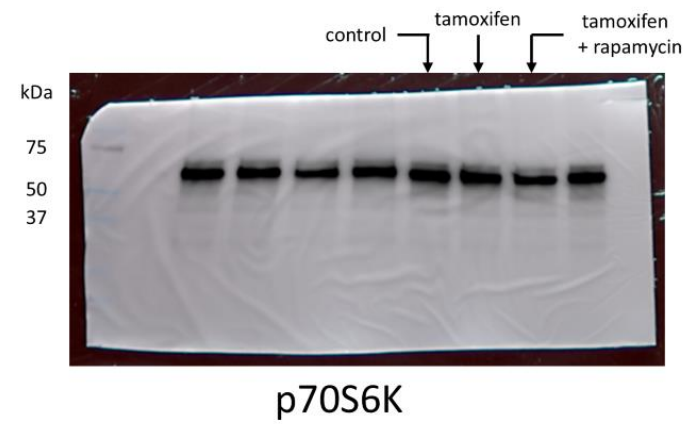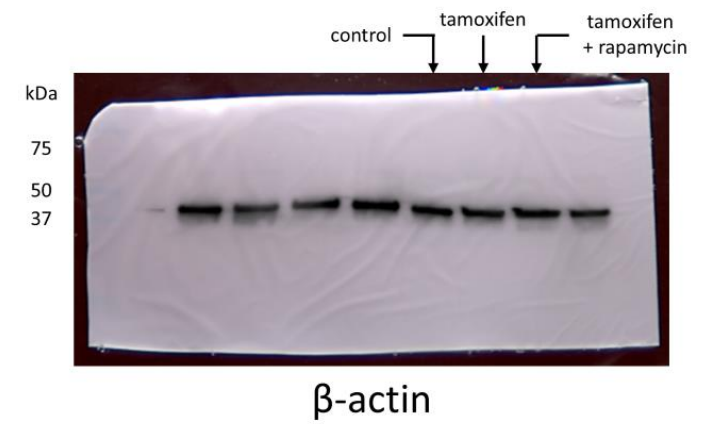

Supplement: Supplementary file 1 — Supplementary Material 1 [file 41598_2025_86586_MOESM1_ESM.pdf]

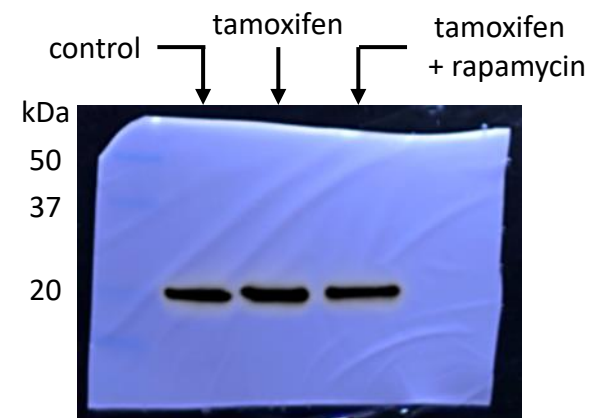

Bax

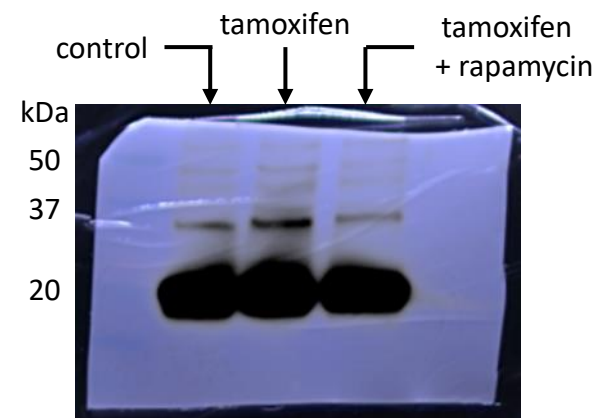

cyclin D1

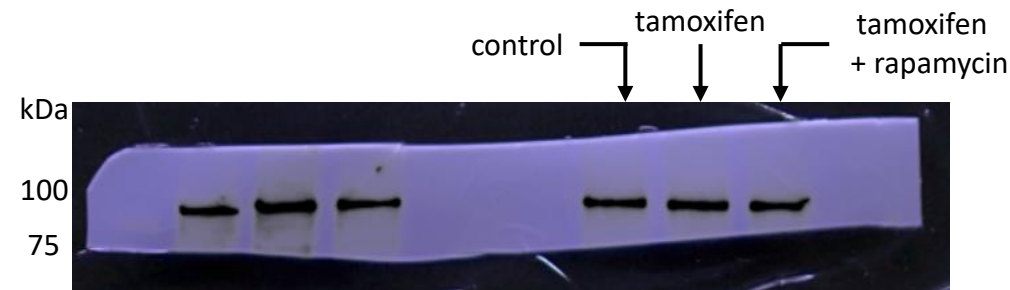

cPARP

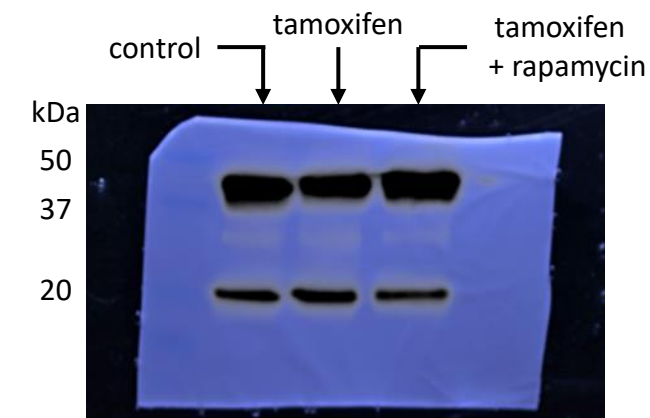

$\beta$ -actin

Supplement: Supplementary file 2 — Supplementary Material 2 [file 41598_2025_86586_MOESM2_ESM.pdf]
